# Supplementary material for: Arsenic contamination of Bangladesh aquifers exacerbated by clay layers
Source: Nat Commun. 2020 May 7;11:2244. doi: 10.1038/s41467-020-16104-z (PMC7205959; doi:10.1038/s41467-020-16104-z)
Supplement: Supplementary file 1 — Supplementary Information [file 41467_2020_16104_MOESM1_ESM.pdf]

Supplementary Information

for

"Arsenic contamination of Bangladesh aquifers exacerbated by clay layers"

by Mihajlov et al.

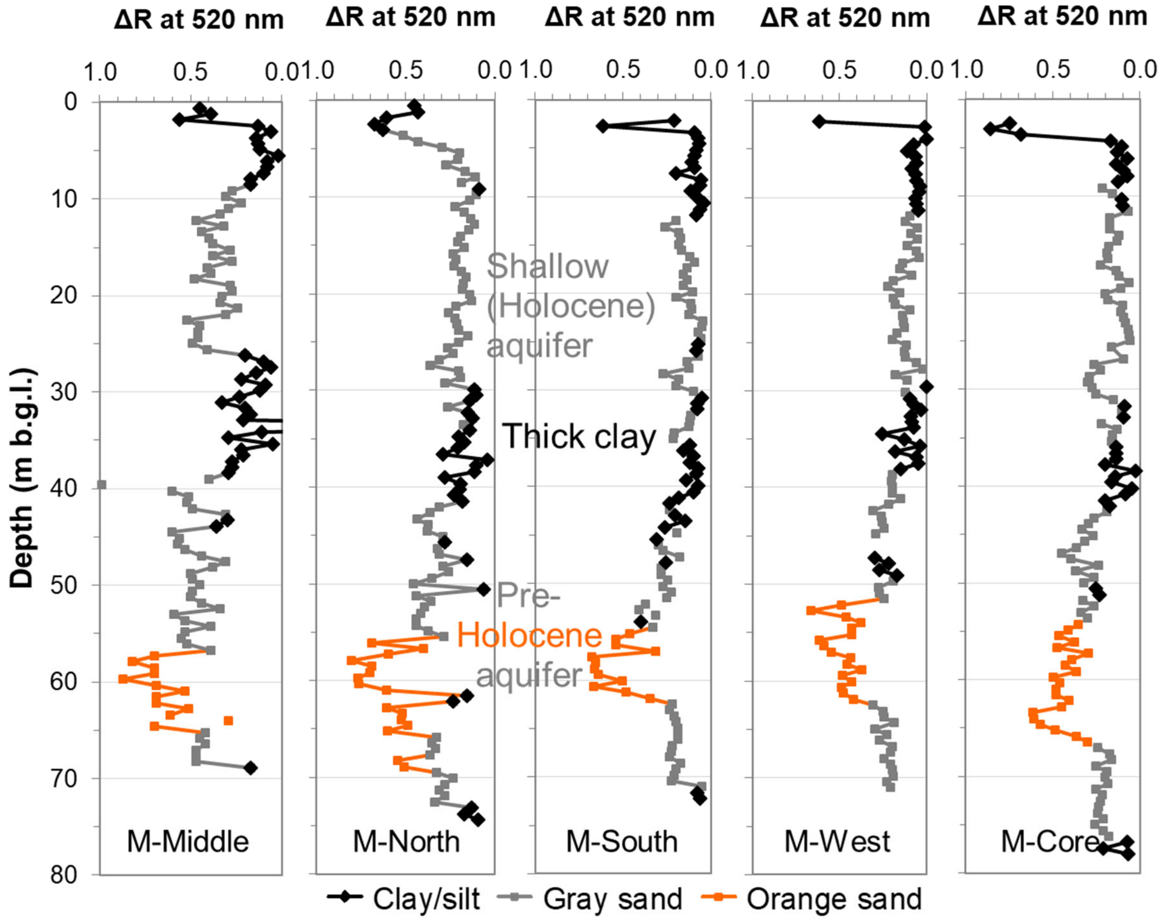

Supplementary Figure 1. Diffuse spectral reflectance between 530 and 520 nm (ΔR) in Site M sediment. Sand color, quantified by ΔR and dictated by Fe speciation, is explicitly displayed to visualize orange and grey sand distribution.

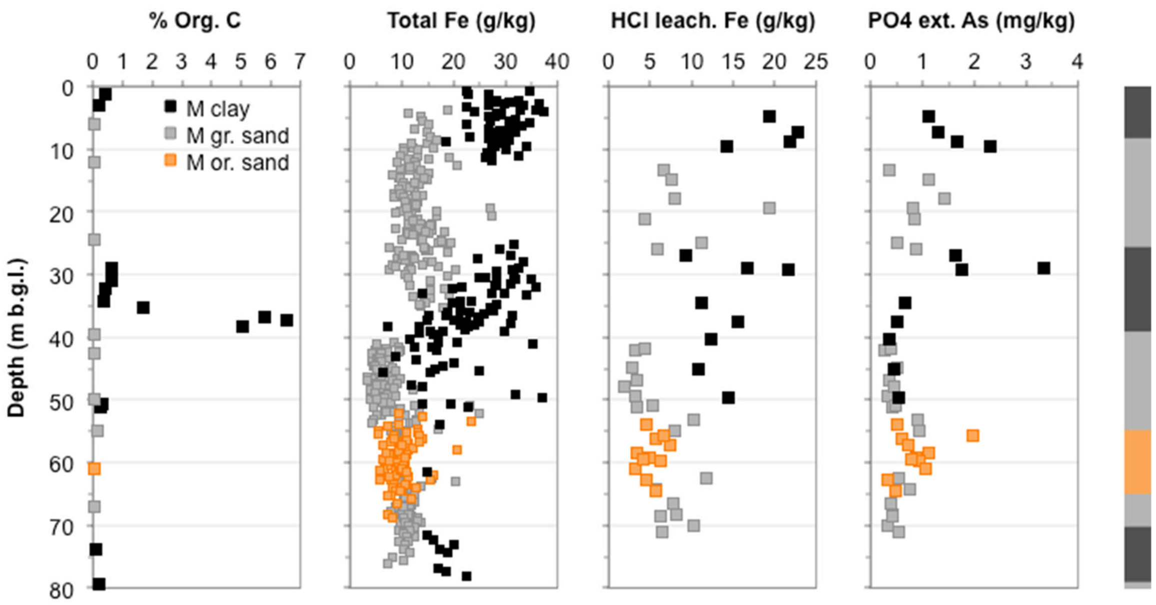

Supplementary Figure 2. Additional site sediment vertical profiles. Displayed profiles include the following (from left to right): percent organic carbon; total iron (Fe) content determined by X-ray fluorescence; Fe extractable by 1N hot HCl; and arsenic (As) extractable by N_2-purged 1M NaH_2PO_4. Sand color, quantified by diffuse spectral reflectance between 530 and 520 nm (ΔR) and dictated by Fe speciation, is explicitly displayed to visualize orange and grey sand distribution. Results from the four multi-level well nest boreholes and the additional coring location are combined in the graphics. A generic site litholog is displayed on the right.

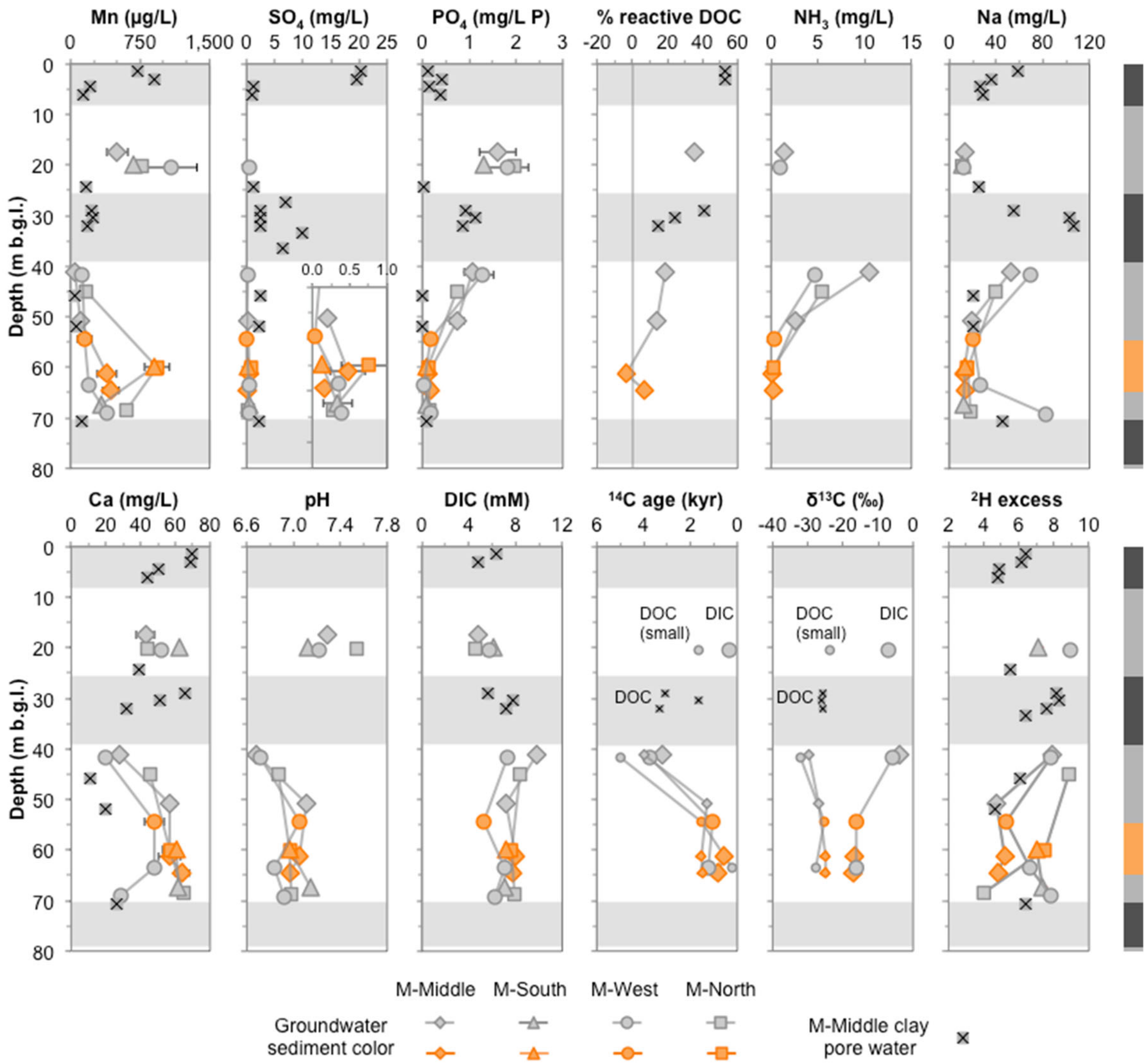

Supplementary Figure 3. Additional vertical profiles of groundwater and clay pore water properties. A generic site litholog is displayed on the right with shading in the panels indicating the extent of major clay/silt layers encountered. Displayed profiles include the following (from left to right, upper panels first): manganese (Mn), sulfate (SO_4), and phosphate (PO_4) concentrations; percent reactive dissolved organic carbon (DOC) as determined in unacidified incubations; ammonia (NH_3), sodium (Na), and calcium (Ca) concentrations; pH values; dissolved inorganic carbon (DIC) concentrations; conventional radiocarbon (^{14}C) ages expressed in thousands of years (kyr) measured on DIC (large symbols) and DOC (small symbols); concentration of carbon-13 ($\delta^{13}C$) measured on DIC (large symbols) and DOC (small symbols); and calculated deuterium (δ^2H) excess ($\delta^2H - 8\times\delta^{18}O$) in water. Percent reactive DOC, NH_3, pH, DIC concentrations, ^{14}C ages, $\delta^{13}C$, δ^2H, and $\delta^{18}O$ are one-time measurements. Analytical errors associated with ^{14}C, $\delta^{13}C$, δ^2H, and $\delta^{18}O$ measurements are smaller than the symbol size. Mn, SO_4, PO_4, Na, and Ca concentrations in groundwater were averaged from discrete samples collected in 2011-2012; at depths where >3 samples were measured, standard deviations are also shown (Mn, SO_4, PO_4, and Ca); Na and most Ca sample standard deviations are smaller than the symbol size.

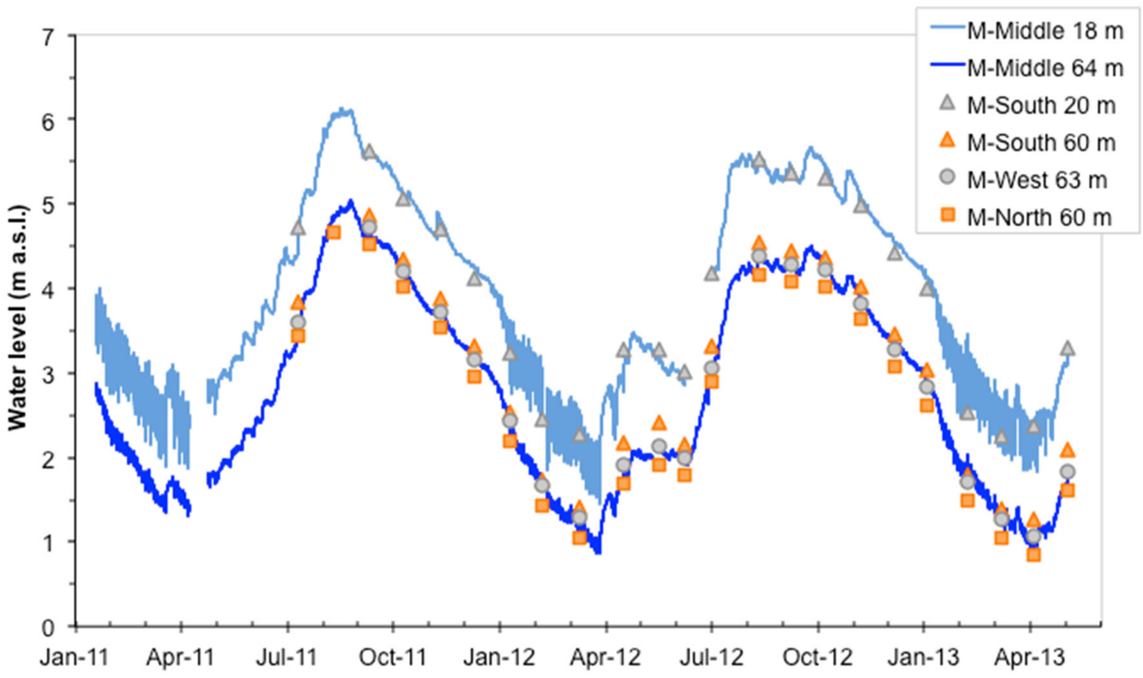

Supplementary Figure 4. **Groundwater elevation hydrographs at study site.** Groundwater elevations above mean sea level across three winter (dry) and two summer (monsoon) seasons. Data from M-Middle location were collected by pressure transducers, whereas discrete-point manual groundwater level measurements are displayed for the remaining locations. Data from the 18 and 20 m depth were collected in the Holocene aquifer above the clay layer; data from 60 to 64 m depth were collected from the orange sand of the pre-Holocene aquifer below the clay layer.

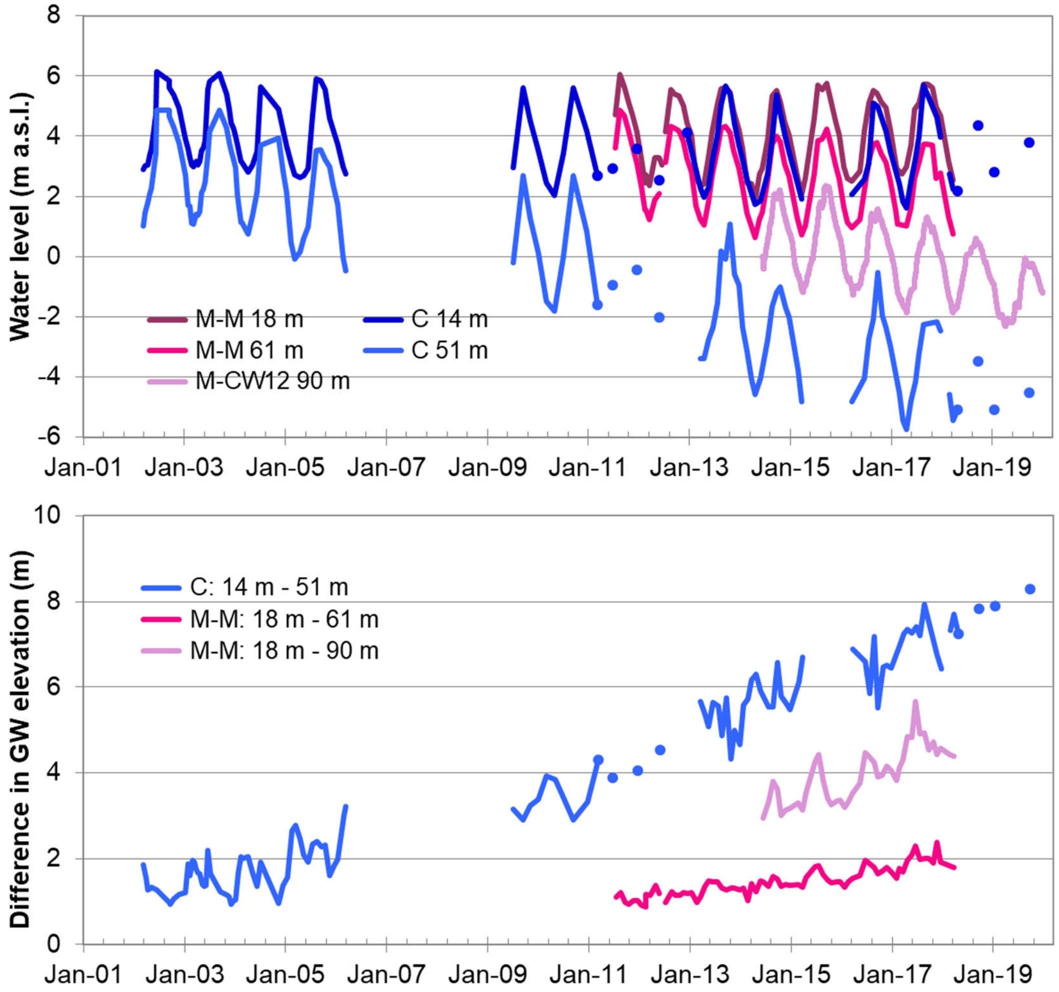

Supplementary Figure 5. **Groundwater elevation hydrographs at study site another site closer to Dhaka.** The longest time series available in the upper panel show monsoonal variations as well as a steady decline in water level in intermediate pre-Holocene aquifer at Site C of Horneman et al. (2004) [7] 2 km closer to Dhaka. The lower panel shows that the vertical head difference relative to a shallow Holocene aquifer increased from 1 m to 8 m over this period. The present study Site M is not as affected by Dhaka pumping [26,27] but the expanding cone of depression still increased the vertical head difference across the 10-15 m thick clay layer to almost 2 m by 2018 (well M-Middle at 61 m depth is shown for illustration and its water levels are similar to those of other depths in the studied pre-Holocene aquifer). The vertical head difference of 5 m was larger for replacement community well CW12 installed at 90 m below the deeper clay layer.

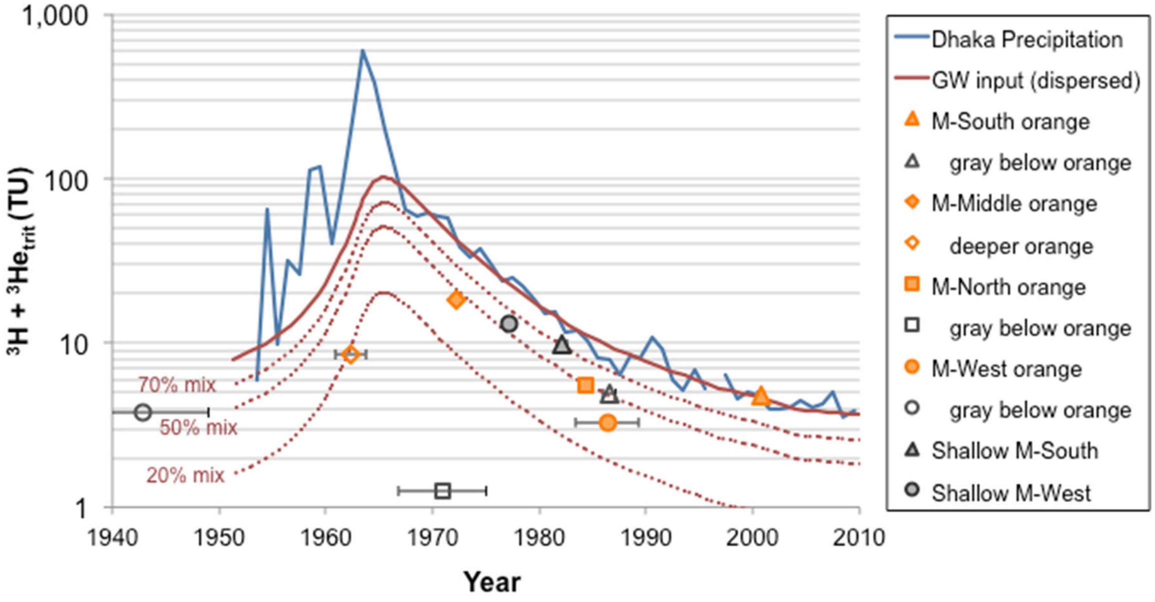

Supplementary Figure 6. Total ^{3}H tracer (sum of measured ^{3}H levels and estimated tritiogenic ^{3}He) plotted against the year of groundwater recharge obtained from ^{3}H/^{3}He age, and compared to the input of ^{3}H from precipitation. This approach to analyzing the ^{3}H/^{3}He ages [4] can demonstrate if a dated groundwater sample was recharged in its entirety in a given year, provided that the sample falls on the ^{3}H input curve from precipitation. In most cases, a water parcel is a mixture of contributions of different ages, the age if the sample reflecting that of the younger, bomb-influenced component [28]. If a sample falls below the input curve, it is a mixture of groundwater of different ages; in the case of binary mixing between ^{3}H-dead (recharged >60 yr ago) and recently recharged groundwater (bomb-influenced), the proportion of recent recharge can be estimated and is shown as percentage of recent recharge by the dashed lines. The estimated input of ^{3}H from Dhaka precipitation, and its dispersed signature in groundwater, were calculated as in Stute [6] and Stute et al. [3]. Shallow here refers samples from the gray, Holocene sand of the shallow aquifer, while the remaining symbols indicate sand color associated with wells screened in the pre-Holocene aquifer.

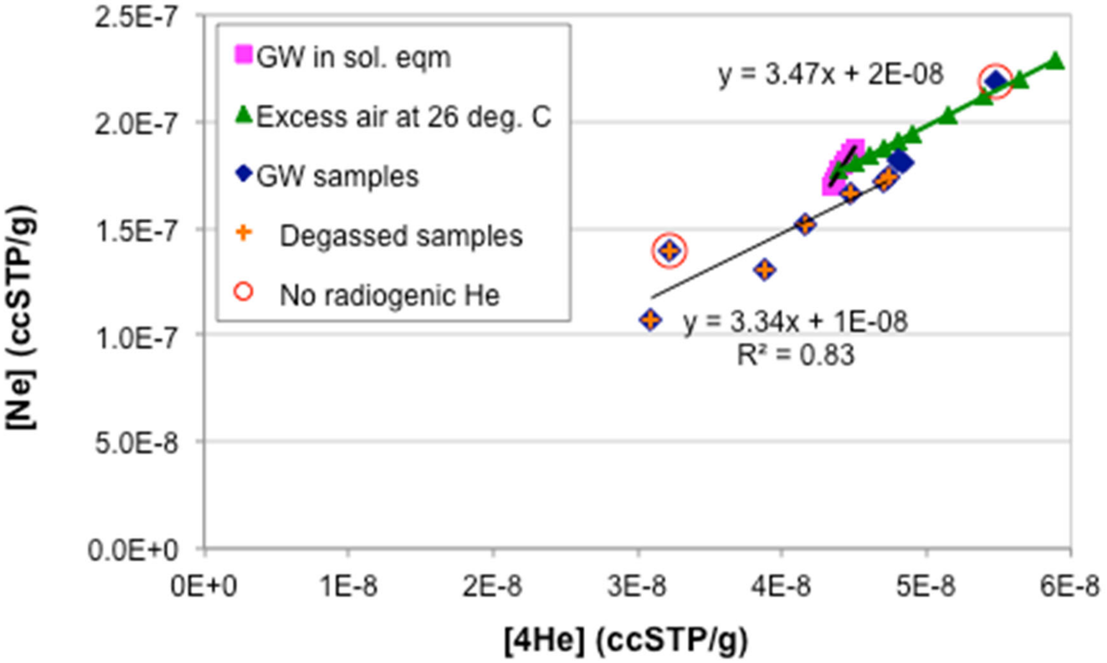

Supplementary Figure 7. A plot of Ne vs. He (as ^{4}He) concentrations in site M groundwater showing the effects of degassing and radiogenic He contributions. Expected concentrations of Ne and He in solubility equilibrium with the atmosphere at a temperature range of 18-32 °C (pink symbols), and those with excess air at 26 °C (additional dissolution of bubbles with atmospheric concentrations, green line), are shown to aid the interpretation of observed groundwater noble gas concentrations (blue squares). Most samples, except the two marked by circles, contain radiogenic He due to the excess of He relative to Ne. The majority of samples (marked by crosses) are also degassed, as their Ne concentrations are lower than those expected in the solubility equilibrium with atmosphere at 26 °C.

Well nest	Depth (m)	C phase	% OC[a]	^{14}C FM[b] ± σ	^{14}C age (kyr)	δ^{13}C (‰PDB)	NOSAMS #[c]
M-N	29.9	clay TOC[d]	0.61	0.5244 ± 0.0027	5.18 ± 0.04	-23.36	OS-103433
M-N	34.1	clay TOC	0.41	0.4120 ± 0.0034	7.12 ± 0.07	-23.68	OS-103479
M-W	36.9	leaves	-	0.3384 ± 0.0015	8.70 ± 0.04	-28.92	OS-91989
M-W	36.9	clay TOC	5.78	0.3399 ± 0.0018	8.67 ± 0.04	-29.01	OS-91887
M-M	37.2	charcoal	-	0.3897 ± 0.0018	7.57 ± 0.04	-28.29	OS-91885
M-N	37.2	wood	-	0.3506 ± 0.0022	8.42 ± 0.05	-29.59	OS-91886
M-N	37.2	clay TOC	6.53	0.3483 ± 0.0015	8.47 ± 0.04	-29.03	OS-91888
M-S	39.3	leaves	-	0.3443 ± 0.0015	8.57 ± 0.04	-30.25	OS-91942
M-N	50.6	clay TOC	0.32	0.2474 ± 0.0014	11.20 ± 0.05	-15.44	OS-92087
M-C	51.2	clay TOC	0.27	0.2243 ± 0.0013	12.00 ± 0.05	-14.94	OS-91944
M-N	73.8	clay TOC	0.10	0.1176 ± 0.0009	17.20 ± 0.07	-21.91	OS-92782
M-C	79.2	clay TOC	0.19	0.0110 ± 0.0022	36.20 ± 1.60	-19.80	OS-91949

[a] % OC Is organic carbon as a percentage of total sediment mass (measured in clay TOC samples only)

[b] FM is "fraction modern". ^{14}C FM of 1 indicates ^{14}C age of 0

[c] NOSAMS # is the tracking number at the National Ocean Science Accelerator Mass-Spectrometer facility

[d] TOC stands for "total organic carbon" i.e. bulk sedimentary OC

n/a = not analyzed

Supplementary Table 1. Radiocarbon dating and ^{13}C analysis of the sediment samples

Well	Depth (m)	Na (mg/L)	±	Mg (mg/L)	±	Si (mg/L)	±	P (mg/L)	±	K (mg/L)	±	Ca (mg/L)	±	Mn (μg/L)	±	Fe (mg/L)	±	As (μg/L)	±
M-M.1	17.5	13	2	14	1	32	3	1.6	0.4	3.2	0.4	43	5	503	116	8.6	2.1	182	20
M-M.4	41.1	52	2	14	0	43	3	1.1	0.2	3.1	0.1	28	2	41	8	10.4	2.4	45	10
M-M.4a	50.9	19	2	24	2	31	2	0.7	0.1	2.6	0.2	57	3	108	30	9.6	2.3	248	37
M-M.5	61.3	12	2	22	3	38	4	0.1	0.1	2.0	0.3	57	6	391	104	0.8	0.9	2	3
M-M.6	64.5	14	1	24	3	37	4	0.2	0.1	2.4	0.3	64	4	434	90	1.0	0.6	5	1
M-N.1	20.1	11	-	15	-	34	-	2.0	-	3.6	-	44	-	768	-	8.4	-	127	-
M-N.4	44.9	40	2	21	1	42	3	0.7	0.1	3.1	0.1	46	1	170	39	8.2	1.1	54	7
M-N.5	60.1	16	1	25	3	46	4	0.1	0.0	2.1	0.5	57	4	935	132	0.8	0.5	3	4
M-N.6	68.6	18	-	28	-	35	-	0.1	-	2.4	-	65	-	597	-	1.6	-	2	-
M-S.1	19.9	11	-	16	-	24	-	1.3	-	3.7	-	62	-	681	-	8.1	-	266	-
M-S.5	59.8	14	-	25	-	34	-	0.1	-	2.2	-	61	-	900	-	0.3	-	3	-
M-S.6	67.4	12	-	26	-	35	-	0.1	-	2.6	-	62	-	333	-	0.7	-	2	-
M-W.1	20.6	12	1	14	1	28	3	1.8	0.5	3.2	0.2	52	4	1087	276	5.9	2.2	298	66
M-W.4	41.8	69	4	9	1	41	7	1.3	0.3	3.4	0.5	20	1	129	54	10.4	4.7	18	3
M-W.5	54.5	20	4	17	1	40	5	0.2	0.1	2.2	0.1	48	6	152	76	2.3	1.3	11	2
M-W.6	63.5	27	-	21	-	43	-	0.0	-	2.0	-	48	-	203	-	0.3	-	2	-
M-W.6a	69.2	82	-	11	-	51	-	0.2	-	2.1	-	29	-	389	-	5.2	-	5	-
M-M CLAY	1.5	59		28		37		0.1		5.2		70		718		4.9		16	
M-M CLAY	3.0	36		28		36		0.4		4.5		68		897		2.0		42	
M-M CLAY	4.6	27		20		42		0.1		5.1		50		213		0.2		47	
M-M CLAY	6.1	29		17		41		0.4		4.9		44		133		0.2		64	
M-M CLAY	24.4	25		15		39		0.0		6.0		39		169		0.1		88	
M-M CLAY	27.4	n/a		n/a		n/a		n/a		n/a		n/a		n/a		n/a		n/a	
M-M CLAY	29.0	56		20		60		0.9		7.6		66		226		3.8		175	
M-M CLAY	30.5	103		18		70		1.1		7.6		51		241		6.5		141	
M-M CLAY	32.0	107		16		63		0.9		7.7		32		186		4.5		95	
M-M CLAY	33.5	n/a		n/a		n/a		n/a		n/a		n/a		n/a		n/a		n/a	
M-M CLAY	36.6	n/a		n/a		n/a		n/a		n/a		n/a		n/a		n/a		n/a	
M-M CLAY	45.7	21		6		18		n/d		2.0		11		48		0.0		3	
M-M CLAY	51.8	21		10		16		n/d		3.2		20		62		0.1		27	
M-M CLAY	70.7	45		13		45		0.1		4.8		26		119		1.4		21	

" - " means std. deviation was not calculated because only 1-3 monthly samples were averaged

n/a = not analyzed

n/d = not detected

Supplementary Table 2. Groundwater and pore water chemical and physical parameters (Part I)

Well	Depth (m)	Sr (μg/L)	±	Ba (μg/L)	±	Cl (mg/L)	±	F (mg/L)	±	SO_4 (mg/L)	±	NH_3 (mg/L N)	Alk. (mEq/L)	pH	eH
M-M.1	17.5	72	6	88	16	7.3	1.2	0.17	0.04	n/d	n/d	1.45	4.3	7.29	-163.5
M-M.4	41.1	92	5	149	16	12.0	0.7	0.25	0.04	n/d	n/d	10.43	6.6	6.68	-122.9
M-M.4a	50.9	161	4	184	13	19.7	1.5	0.29	0.04	0.20	-	2.62	6.0	7.11	-171.3
M-M.5	61.3	151	6	129	8	8.3	1.0	0.41	0.04	0.47	0.23	0.08	6.6	7.05	-54.6
M-M.6	64.5	179	14	162	22	7.0	0.6	0.44	0.05	0.16	-	0.22	6.2	6.97	-69.6
M-N.1	20.1	69	-	97	-	8.4	-	0.17	-	n/d	n/d	n/a	4.3	7.54	-165.9
M-N.4	44.9	149	6	185	13	8.3	0.4	0.24	0.02	n/d	n/d	5.38	6.4	6.87	-112.0
M-N.5	60.1	162	6	154	15	7.8	0.7	0.30	0.04	0.76	0.38	0.21	6.1	6.97	-32.8
M-N.6	68.6	176	-	143	-	6.7	-	0.32	-	0.27	-	n/a	6.4	6.98	-66.8
M-S.1	19.9	94	-	113	-	5.7	-	0.19	-	n/d	n/d	n/a	5.2	7.12	-160.6
M-S.5	59.8	n/a	n/a	35	-	7.9	-	0.40	-	0.13	-	n/a	5.7	6.97	-67.8
M-S.6	67.4	168	-	93	-	8.0	-	0.45	0.03	0.34	0.20	n/a	6.1	7.15	-78.0
M-W.1	20.6	89	13	69	15	3.6	0.8	0.27	0.02	0.44	-	0.92	5.0	7.22	-168.2
M-W.4	41.8	59	2	140	24	18.8	1.4	0.28	0.07	0.12	-	4.7	5.1	6.72	-90.0
M-W.5	54.5	126	8	182	13	9.7	1.3	0.30	0.04	0.04	-	0.33	4.4	7.05	-82.0
M-W.6	63.5	n/a	n/a	97	-	6.3	-	0.35	-	0.36	-	n/a	5.3	6.83	-50.3
M-W.6a	69.2	60	-	60	-	6.4	0.3	0.29	0.07	0.39	-	n/a	4.9	6.92	-68.0
M-M CLAY	1.5	94		228		47.3		0.18		20.36					
M-M CLAY	3.0	88		139		87.2		0.26		19.52					
M-M CLAY	4.6	81		126		44.7		0.20		1.17					
M-M CLAY	6.1	81		113		28.5		0.22		0.90					
M-M CLAY	24.4	74		111		13.0		0.13		1.30					
M-M CLAY	27.4	n/a		n/a		34.7		0.21		7.00					
M-M CLAY	29.0	126		173		26.6		0.18		2.43					
M-M CLAY	30.5	107		145		29.8		0.19		2.35					
M-M CLAY	32.0	80		137		31.7		0.18		2.54					
M-M CLAY	33.5	n/a		n/a		63.2		0.19		9.95					
M-M CLAY	36.6	n/a		n/a		52.8		0.27		6.46					
M-M CLAY	45.7	33		67		27.2		0.24		2.43					
M-M CLAY	51.8	57		95		59.8		0.25		2.31					
M-M CLAY	70.7	81		162		25.8		0.22		2.14					

" - " means std. deviation was not calculated because only 1-3 monthly samples were averaged

n/a = not analyzed

n/d = not detected

Supplementary Table 2. Groundwater and pore water chemical and physical parameters (Part II)

Well	Depth (m)	Cond. (mS/cm)	Temp. (C)	DIC (mM)[a]	DOC (mM)[b]	% reactive DOC	$\delta^{18}O$ (‰)	± σ	$\delta^{2}H$ (‰)	± σ
M-M.1	17.5	0.459	n/a	4.8	0.15	35.1	n/a	n/a	n/a	n/a
M-M.4	41.1	0.655	26.34	9.9	0.45	18.7	-4.43	0.02	-27.54	0.03
M-M.4a	50.9	0.679	26.37	7.1	0.41	14.0	-3.26	0.04	-21.32	0.08
M-M.5	61.3	0.612	26.52	8.0	0.17	-3.5	-3.16	0.01	-20.05	0.09
M-M.6	64.5	0.634	26.63	7.8	0.20	6.8	-3.20	0.04	-20.73	0.02
M-N.1	20.1	0.425	n/a	4.6	n/a	n/a	n/a	n/a	n/a	n/a
M-N.4	44.9	0.668	26.30	8.4	0.35	n/a	-4.49	0.02	-27.04	0.10
M-N.5	60.1	0.607	26.39	7.6	0.15	n/a	-3.09	0.02	-17.20	0.07
M-N.6	68.6	0.635	26.50	7.9	n/a	n/a	-3.15	0.03	-21.23	0.02
M-S.1	19.9	0.564	26.20	6.1	n/a	n/a	-2.39	0.02	-12.02	0.07
M-S.5	59.8	0.572	26.16	7.1	n/a	n/a	-3.02	0.02	-17.15	0.02
M-S.6	67.4	0.611	26.18	7.1	n/a	n/a	-3.44	0.01	-20.13	0.05
M-W.1	20.6	0.514	26.46	5.7	0.20	n/a	-2.90	0.01	-14.25	0.04
M-W.4	41.8	0.604	26.69	7.3	0.30	n/a	-4.41	0.01	-27.45	0.06
M-W.5	54.5	0.472	26.43	5.3	0.14	n/a	-3.77	0.04	-24.86	0.10
M-W.6	63.5	0.538	26.56	7.1	n/a	n/a	-3.90	0.03	-24.55	0.01
M-W.6a	69.2	0.501	26.65	6.2	n/a	n/a	-3.89	0.04	-23.32	0.09
M-M CLAY	1.5			6.3	0.60	52.9	-1.95	0.03	-9.17	0.13
M-M CLAY	3.0			4.8	0.57	52.8	-2.14	0.03	-10.98	0.02
M-M CLAY	4.6			n/a	0.69	n/a	-1.46	0.06	-6.82	0.24
M-M CLAY	6.1			n/a	0.60	n/a	-1.47	0.04	-6.91	0.23
M-M CLAY	24.4			n/a	0.72	n/a	-1.71	0.02	-8.12	0.08
M-M CLAY	27.4			n/a	n/a	n/a	n/a	n/a	n/a	n/a
M-M CLAY	29.0			5.7	1.75	41.1	-4.15	0.06	-25.01	0.19
M-M CLAY	30.5			7.7	1.80	24.1	-4.45	0.05	-27.35	0.20
M-M CLAY	32.0			7.2	1.88	14.8	-4.08	0.02	-25.07	0.23
M-M CLAY	33.5			n/a	n/a	n/a	-3.06	0.01	-18.12	0.07
M-M CLAY	36.6			n/a	n/a	n/a	n/a	n/a	n/a	n/a
M-M CLAY	45.7			n/a	n/a	n/a	-3.29	0.01	-20.27	0.14
M-M CLAY	51.8			n/a	n/a	n/a	-2.61	0.02	-16.20	0.06
M-M CLAY	70.7			n/a	1.21	n/a	-2.97	0.07	-17.40	0.09

[a] DIC values for site M groundwater were calculated from pH and alkalinity measured simultaneously in the field.
DIC in M1 clay pore water was measured on the Shimadzu carbon analyzer. DIC at site T was measured by NOSAMS

[b] DOC values were measured on the Shimadzu carbon analyzer, except at site T (measured by NOSAMS)
n/a = not analyzed

Supplementary Table 2. Groundwater and pore water chemical and physical parameters (Part III)

Well	Depth (m)	DIC ^{14}C Fm[a]	± σ	DIC ^{14}C age (yr)	± σ	DIC $\delta^{13}C$ (‰)	NOSAMS #[b]	DOC ^{14}C FM	± σ	DOC ^{14}C age (yr)	± σ	DOC $\delta^{13}C$ (‰)	NOSAMS #
M-W.1	20.6	0.9607	0.0034	320	30	-7.1	OS-89968	0.8114	0.0030	1,680	30	-23.9	OS-102042
M-M CLAY	29.0	n/a	n/a	n/a	n/a	n/a	n/a	0.6821	0.0027	3,070	30	-25.8	OS-101625
M-M CLAY	30.5	n/a	n/a	n/a	n/a	n/a	n/a	0.8147	0.0024	1,650	25	-26.0	OS-101626
M-M CLAY	32.0	n/a	n/a	n/a	n/a	n/a	n/a	0.6629	0.0018	3,300	20	-25.7	OS-102082
M-M.4	41.1	0.6705	0.0025	3,210	30	-4.0	OS-90067	0.6110	0.0032	3,960	40	-29.7	OS-101494
M-W.4	41.8	0.6278	0.0022	3,740	25	-5.7	OS-89969	0.5369	0.0023	4,990	35	-32.2	OS-102041
M-M.4a	50.9	n/a	n/a	n/a	n/a	n/a	n/a	0.8521	0.0021	1,280	20	-26.8	OS-101813
M-W.5	54.5	0.8774	0.0027	1,050	25	-16.1	OS-90175	0.8289	0.0019	1,510	20	-25.3	OS-102065
M-M.5	61.3	0.9312	0.0030	570	25	-16.6	OS-90066	0.8259	0.0021	1,540	20	-24.9	OS-101857
M-W.6	63.5	0.8592	0.0028	1,220	25	-16.2	OS-90173	0.9748	0.0027	205	20	-27.6	OS-102111
M-M.6	64.5	0.9018	0.0034	830	30	-17.1	OS-90176	0.8304	0.0027	1,490	25	-25.0	OS-101682

[a] FM is "fraction modern". ^{14}C FM of 1 indicates ^{14}C age of 0

[b] NOSAMS # is the tracking number at the National Ocean Science Accelerator Mass-Spectrometer facility

n/a = not analyzed

Supplementary Table 3. Radiocarbon dating and ^{13}C analysis of dissolved inorganic and organic carbon (DIC and DOC) in groundwater

Well ID	Depth	^3H	± 1σ	^4He	Ne	δ^3He [a]	^3H + ^3He	$^3H/^3He$ age[b]	± 1σ corr.[b]	± 1σ anal.[c]
	m	TU	TU	x10^{-8} ccSTP g^{-1}	x10^{-8} ccSTP g^{-1}	%	TU	years	years	years
M-M.1	17.5	n/a	n/a	n/a	n/a	n/a	n/a	n/a	n/a	n/a
M-M.4	41.1	0.09	0.03	n/a	n/a	n/a	n/a	n/a	n/a	n/a
M-M.4a	50.9	1.61	0.05	n/a	n/a	n/a	n/a	n/a	n/a	n/a
M-M.5	61.3	2.04	0.06	4.47	16.64	50.1	18.2	39.2	1.9	0.4
M-M.6	64.5	0.54	0.03	4.73	17.40	20.1	8.4	49.1	1.4	1.0
M-N.1	20.1	n/a	n/a	n/a	n/a	n/a	n/a	n/a	n/a	n/a
M-N.4	44.9	0.06	0.03	n/a	n/a	n/a	n/a	n/a	n/a	n/a
M-N.5	60.1	1.22	0.05	4.84	18.03	7.2	5.5	27.0	-	0.6
M-N.6	68.6	0.13	0.03	4.70	17.20	-3.5	1.3	40.5	1.8	4.2
M-S.1[d]	19.9	1.89	0.06	5.47	21.92	24.3	9.7	29.3	-	0.4
M-S.5	59.8	2.68	0.06	3.10	10.75	0.2	4.8	10.6	0.0	0.3
M-S.6	67.4	1.21	0.05	3.89	13.06	0.7	4.9	24.9	0.1	0.6
M-W.1	20.6	1.94	0.05	4.81	18.17	34.4	13.1	34.2	-	0.4
M-W.4	41.8	-0.01	0.03	n/a	n/a	n/a	n/a	n/a	n/a	n/a
M-W.5[d]	54.5	0.81	0.04	3.21	13.91	8.5	3.3	25.0	3.0	0.6
M-W.6	63.5	0.08	0.03	4.15	15.22	5.6	3.7	68.6	1.2	6.2
M-W.6a	69.2	0.18	0.03	n/a	n/a	n/a	n/a	n/a	n/a	n/a

[a] δ^3He is a % difference from the atmospheric $^3He/^4He$ ratio

[b] The $^3H/^3He$ age is a mean of three ages calculated from degassing corrections, and 1σ corr. is the standard deviation of the mean. Samples M-N.5, M-S.1, and M-W.1 were not degassed (-)

[c] Standard error propagated from the analytical measurements alone, excluding degassing corrections

[d] Samples M-S.1 and M-W.5 were not corrected for radiogenic He contribution to the $^3He/^4He$ ratio

Supplementary Table 4. $^3H/^3He$ dating parameters and ages calculated using radiogenic He correction (except where noted)

Well ID	Depth	$^3H/^3He$ age[a]	± 1σ corr.[a]	$^3H/^3He$ age[b]	± 1σ anal.[c]
	m	years	years	years	years
M-M.1	17.5	n/a	n/a	n/a	n/a
M-M.4	41.1	n/a	n/a	n/a	n/a
M-M.4a	50.9	n/a	n/a	n/a	n/a
M-M.5	61.3	37.4	2.1	35.2	0.5
M-M.6	64.5	44.9	1.8	42.5	1.0
M-N.1	20.1	n/a	n/a	n/a	n/a
M-N.4	44.9	n/a	n/a	n/a	n/a
M-N.5	60.1	19.3	-	19.3	0.8
M-N.6	68.6	ND	ND	ND	ND
M-S.1[d]	19.9	29.3	-	29.3	0.5
M-S.5	59.8	2.8	0.1	0.2	0.6
M-S.6	67.4	7.3	0.4	2.2	1.1
M-W.1	20.6	32.0	-	32.0	0.5
M-W.4	41.8	n/a	n/a	n/a	n/a
M-W.5[d]	54.5	25.0	3.0	18.9	0.9
M-W.6	63.5	57.5	2.3	50.5	6.1
M-W.6a	69.2	n/a	n/a	n/a	n/a

[a] The $^3H/^3He$ age is a mean of three ages calculated from degassing corrections, and 1σ corr. is the standard deviation of the mean. Samples M-N.5, M-S.1, and M-W.1 were not degassed (-)

[b] The $^3H/^3He$ age calculated by assuming degassing at time of recharge

[c] Standard error propagated from the analytical measurements alone, excluding degassing corrections

ND = not determinable because of a low measured $^3H/^3He$ ratio

n/a = not analyzed

Supplementary Table 5. $^3H/^3He$ ages and errors calculated without radiogenic He corrections, assuming degassing occurred either at time of sampling (with a range of corrections) or at time of recharge.

Supplementary Note 1

^{3}H tracer and $^{3}H/^{3}He$ dating of groundwater: Elevated levels of tritium (^{3}H), a telltale sign of recharge with young groundwater, were found in the pre-Holocene aquifer at site M, at depths of >50 m bgl, and that despite the presence of a 10-15 m thick clay layer at the site (Fig. 3b and Supplementary Table 4). The amount of ^{3}H decreased along the S-N transect at site M, while well nest M-West appeared isolated from the supply of the radioactive H isotope. Tritium values at each M well nest were the highest in the orange sand layer, and decreased in the grey layers at top and bottom of the aquifer, but the upper pre-Holocene aquifer immediately beneath the thick clay layer was markedly devoid of detectable ^{3}H. The one exception is well M-M.4a from 51m depth that was close to the bottom of the grey layer and contained ^{3}H, likely because it is at an interface between the slowly and rapidly recharged portions of the pre-Holocene aquifer. This is one of the wells that was monitored for a decade and shows increasing As concentrations. The ^{3}H data obtained in 2011 cannot rule out a contribution of a <10% contribution of groundwater from 51 m (where well M-M.4a is screened) to the 41 m depth interval tapped by well M-M4 which has also shown increasing As concentrations since 2011. Despite the downward vertical hydraulic gradient at the site, the apparent vertical mixing could be due to groundwater flow dispersion and the fact that the two screens are 1.5 m long.

The concentrations of ^{3}H and its radioactive daughter ^{3}He were used to estimate the age of the intruding young groundwater by calculating the time elapsed since recharge ($^{3}H/^{3}He$ age, Fig. 3c, and Supplementary Fig. 6 and Tables 4 and 5). In order to calculate the amount of ^{3}He contributed by the radioactive decay of ^{3}H ("tritiogenic ^{3}He" or "$^{3}He_{trit}$"), the concentrations of noble gases He (as ^{4}He) and Ne were plotted against each other to account for different pools of He (Supplementary Fig. 7). The recharge temperature for all samples was assumed to be 26 °C, close to the current average shallow groundwater temperatures at the site (Supplementary Table 2). The concentrations of He and Ne exceeded the contributions of the solubility equilibrium with the atmosphere due to excess air formation (trapped bubbles) by a large amount only in sample M-S.1, and slightly in samples M-W.1 and M-N.5. Samples M-W.1 and M-N.5 also had excess He compared to the excess air curve at 26 °C, which was attributed to the production of radiogenic He. The remaining samples were degassed compared to the solubility equilibrium to various degrees: three samples had a slight deficit of Ne (<10%), while the remaining four samples lost up to 30% of the He and 40% of the Ne expected at equilibrium.

For the ages presented in Fig. 3c and Supplementary Fig. 6, the degassing loss was assumed to have occurred at sampling, without a significant amount of fractionation between He and Ne and their isotopes. The concentrations of He were then back corrected for the degassed samples to the values they would have had (1) at solubility equilibrium, (2) with a slight excess air amount as in sample M-N.5, or (3) with a larger amount of excess air as in sample M-S.1. Six of the degassed samples (all except M-W.5) were also assumed to have a contribution of radiogenic He similar to that of the samples M-N.5 and M-W.1 because they had larger deficits of Ne than He, whereas loss due to degassing would have favored larger deficits of He [1,2]. The $^{3}He/^{4}He$ ratio in solubility equilibrium with the

atmosphere (1.36×10^{-6}), and where necessary the ratio in the atmosphere (1.384×10^{-6}) and in the radiogenic He (crustal 2×10^{-8}), were then utilized to calculate the amount of $^{3}He_{trit}$ and the $^{3}H/^{3}He$ ages. In case of the degassed samples, the average of the three corrections for degassing was reported. The standard deviations of the three ages resulting from the back corrections (σ corr.), as well as the standard deviations in ages resulting from the analytical errors alone (σ anal.), were reported in Supplementary Table 4; the larger of the two errors in individual samples was used in Fig. 3c and Supplementary Fig. 6.

The calculation presented above was also carried out, as a quality control, without radiogenic He corrections in the 8 samples with probable radiogenic He contributions, assuming either (1) the three scenarios of degassing at the time of sampling, or (2) that degassing occurred at the time of recharge by exchange with atmosphere. The resulting age averages and error estimates from these additional calculations are presented in Supplementary Table 5.

The apparent $^{3}H/^{3}He$ ages of site M samples in 2011 (Fig. 3c) ranged from 11 to 69 years. Samples from ~20 m bgl in the shallow aquifer had a constant age of roughly 30 years. In the pre-Holocene aquifer, however, the samples collected from the orange sand layer had younger ages than those from the deeper grey sand layer, providing evidence that flow in the middle of the aquifer (orange sand) is more vigorous. The youngest age of ~11 years was found in well M-S.5, screened in the orange sand at the southern edge of the site. Although the horizontal hydraulic gradient in the orange sand is directed toward the north (Fig. 3a and Supplementary Fig. 4), there is no evidence of a simple south-to-north flowpath along the orange sand layer at the site, as the $^{3}H/^{3}He$ age increased to ~39 years at well nest M-Middle, but leveled off at ~25-27 years at well nests M-North and M-West. The above interpretation of $^{3}H/^{3}He$ ages only holds true either if the sampled groundwater was entirely recharged at one time point since the nuclear bomb testing started, or if the groundwater is a binary mix of recently recharged groundwater containing bomb-produced ^{3}H and older, pre-bomb groundwater. In the latter case, the $^{3}H/^{3}He$ ages only apply to the fraction of groundwater contributed by recent recharge.

The actual amount of mixing or dispersion in groundwater samples can be estimated by adding the measured ^{3}H to the estimated $^{3}He_{trit}$, their sum acting as a "stable ^{3}H" conservative tracer [3-5]. The initial input of ^{3}H from the atmosphere by precipitation was estimated for Bangladesh, and its signal in groundwater predicted using typical rates of dispersion, by Stute [6] and Stute et al. [3]. The comparison of $^{3}H+^{3}He_{trit}$ estimates at site M to the groundwater input curve (Supplementary Fig. 6) showed that only the most recently recharged sample at M-S.5 (orange sand at southern edge of the site) fell on the input curve, and thus was not mixed with the pre-bomb groundwater. High percentages of admixed younger groundwater were found in the shallow groundwater (50-70%), and in the orange sand layer along the south-to-north transect (nests M-Middle and M-North, ~50%). Lower contributions of recent recharge were observed in the deeper grey layer of the pre-Holocene aquifer compared to the orange sand, and well nest M-West at both pre-Holocene aquifer depths received less young groundwater input than the wells along the south-to-north transect.

Supplementary Note 2

Reducing Conditions in the Clay Pore water and Groundwater: Concentrations of dissolved Fe of >5 mg/L and the higher proportion (>0.5) of Fe(II) within the total Fe extractable by 1N hot HCl [7] from the grey sand under the clay layer, compared to mostly <1 mg/L dissolved Fe and <0.5 Fe(II) fraction further down in the orange sand, indicate strongly reducing conditions in the grey sands that most likely caused the release of As to groundwater (Fig. 3e,f). Higher concentrations of phosphate (PO_4) and ammonia (NH_3) in the grey sands below the clay layer (Supplementary Fig. 3) are also consistent with advanced dissimilatory Fe reduction and OM decomposition at this depth [8-11]. In contrast, the higher concentrations of manganese (Mn) and detectable sulfate (SO_4) in the orange sand below (Supplementary Fig. 3) suggest more oxidizing conditions that could suppress the release of Fe and As to groundwater [12].

The concentrations of dissolved Fe, As, and PO_4 in the pore water of the thick clay layer are similar to those in the grey pre-Holocene sand below the clay (Fig. 3e,f and Supplementary Fig. 3). As such, the clay layer could be a direct source of As into the underlying pre-Holocene aquifer by advection or dispersion of the pore water. However, even if water extracted from the clay by squeezing also contains As and Fe at levels comparable to those in the grey sand below the clay, we focus on the elevated DOC content of clay water (Fig. 3g) because that is the constituent that can drive the reductive dissolution of Fe oxides and because only a portion of the groundwater beneath the clay layer is derived directly from the clay porewater (see discussion below). Simple incubation experiments indicate that a considerable proportion of the DOC, especially that squeezed from the clay layer, decomposes within a month (Supplementary Fig. 3) and can therefore be assumed to be reactive [13].

Supplementary Note 3

Estimate of the Contribution of Clay Pore Water to Upper pre-Holocene Aquifer Using Chloride as a Tracer: An independent check of the contribution of clay pore water and clay-derived DOC to the upper pre-Holocene aquifer can be provided by using chloride (Cl) dissolved in groundwater as an inert anionic tracer of water movement as Cl is particularly concentrated in the thick clay pore water (Fig. 3h). Besides DOC and dissolved As, clays also often host elevated concentrations of anions (e.g., chloride) due to a combination of factors such as high organic matter content, low flushing rates after deposition often in deltaic settings, and high porosity and sorption capacity [14-17]. In the pre-Holocene aquifer, the highest levels of Cl of 8-20 mg/L are found in groundwater from the upper grey layer, indicating that clay pore water likely contributes to anionic composition of groundwater immediately below. Groundwater from the rest of the pre-Holocene aquifer has Cl concentrations of 6-9 mg/L, i.e. more similar to those found in the shallow aquifer (Fig. 3h). Based on the Cl concentrations of 53-63 mg/L at the bottom half of the clay layer, it is estimated that the clay pore water contributes approximately 20% of the total groundwater present in the upper pre-Holocene aquifer layer.

An important implication of the estimated fraction of clay pore water contributed to the groundwater immediately below the clay is that the similar concentrations of dissolved As, Fe, and PO_4 observed in both the clay and the grey sand underneath (Fig. 3e,f and Supplementary Fig. 3) cannot be explained by direct transport of these solutes out of the clay layer. When transported into the pre-Holocene aquifer, those solutes would be subject to both the dilution and adsorption. Thus, to make up the difference, reduction of labile Fe oxides fueled by clay-derived DOC is required to release additional Fe, as well as As and PO_4 adsorbed on the Fe oxide mineral phases.

Supplementary Note 4

Isotopic Signatures of Clay Pore Water and Groundwater: Environmental tracer signatures in pore water extracted from the upper half of the clay layer are similar to those of groundwater in the grey sands directly underneath, and distinct from those of groundwater within the orange sands further down (Fig. 3d and Supplementary Fig. 3), which suggests either the clay pore water leakage into the upper pre-Holocene aquifer or a similar recharge history of the two units. Stable isotopes of the water molecule, $^{18}O/^{16}O$ ($\delta^{18}O$) and $^{2}H/^{1}H$ ($\delta^{2}H$), have a more depleted signature in both the pore water from the upper half of the clay layer and the groundwater from upper pre-Holocene aquifer (Fig. 3d and Supplementary Table 2), and the two layers also have a similarly high deuterium (^{2}H) excess (Supplementary Fig. 3). In contrast, the orange and lower grey sand layers of the pre-Holocene aquifer host groundwater with $\delta^{18}O$ and $\delta^{2}H$ values closer to those from the shallow aquifer.

These observations made from the stable isotopes are consistent with the measured ^{3}H concentrations in the pre-Holocene aquifer and the implied groundwater residence times. The concentrations of ^{3}H in groundwater from the upper pre-Holocene aquifer are near or below the detection limit, indicating that recharge at this depth is >60 years old and may contain a fraction of older, ^{3}H-free clay pore water. Because of the similar $\delta^{18}O$ and $\delta^{2}H$ in the available samples from the capping clay and the grey sands below, and because the samples from the bottom half of the clay layer could not be collected, a relative amount of the clay pore water contribution in the upper pre-Holocene aquifer cannot be estimated from these tracers; the isolation of this part of the aquifer from recent recharge may have retained its stable isotopic signature distinct from that of the shallow aquifer and the orange sand below. The more negative $\delta^{18}O$ and $\delta^{2}H$ values in the clay and in the upper pre-Holocene aquifer (relative to the shallow aquifer and deeper in the pre-Holocene aquifer) and the consistently high ^{2}H excess in these layers may reflect distinct climatic conditions when these layers with the sluggish flow were recharged. Further interpretation of the paleoclimate conditions cannot be made based on this site alone and multiple factors may affect the complex water stable isotope signature. The orange sands in the pre-Holocene aquifer contain ^{3}H from recent recharge and $\delta^{18}O$ and $\delta^{2}H$ values at that depth are, accordingly, more similar to that of the shallow aquifer, although the ^{2}H excess in this layer is variable.

The radiocarbon ages of DOC from the grey sands in the upper pre-Holocene aquifer are close to DOC radiocarbon ages in the capping clay layer and 2-4,000 years greater than

those in deeper layers of the pre-Holocene aquifer (Supplementary Fig. 3 and Table 3). This observation is in agreement with the contributions of older, clay DOC along the thick clay/sand interface, and more vigorous lateral recharge that contributes bomb radiocarbon within orange sand along the middle of the aquifer.

Although radiocarbon ages of DIC were not measured in the thick clay layer, those measured in the upper grey sands of the pre-Holocene aquifer are close in age to the resident DOC and older than the radiocarbon ages of DIC deeper in the pre-Holocene aquifer (Supplementary Fig. 3 and Table 3). Carbon stable isotope ($\delta^{13}C$) signature of the DIC is particularly enriched (more positive) in the grey sand directly beneath the thick clay, suggesting that methanogenesis may be occurring in this layer. The well-advanced reduction in the grey sand below the clay layer is also characterized by the minimum Eh values within the aquifer (Supplementary Table 2). Methanogens preferentially use ^{12}C for methane production, leaving behind ^{13}C-enriched DIC, and their presence has been implicated in highly reduced, As-rich shallow groundwater of Bangladesh [18]. A similar ^{14}C and ^{13}C signature of DIC in the grey sand below the thick clay could have been produced by dissolution of radiocarbon-free carbonates; however, this scenario is less likely given the dissolved Ca and pH minimum in this layer of the aquifer (Supplementary Fig. 3), and that no sedimentary inorganic carbon could be measured (<0.01 wt%).

Supplementary Note 5

Estimate of the Advective and Diffusive Flux of DOC into the Upper pre-Holocene Aquifer and the Amount of Iron Reduction: In this section, the extent of Fe reduction is estimated based on advection and molecular diffusion of DOC at the aquifer-aquitard interface, which is a prerequisite for the in-situ release of As in the pre-Holocene aquifer.

The advective flow component of groundwater from the clay can be calculated using Darcy's flux, which is expressed as: $Q = -K\frac{dh}{dx}A$; where, A = cross-section area (assuming 1 m^2); $\frac{dh}{dx}$ = hydraulic gradient, and K (vertical hydraulic conductivity) = 10^{-9}-10^{-7} m/sec based on permeameter/falling head tests from similar materials in Zheng et al. [19]. If we consider a vertical hydraulic head difference of 1 m across the 10 m thick clay (Fig. 3a), then $\frac{dh}{dx} = 0.1$. Therefore, the vertical advective flux of groundwater per square m would be 10^{-10}-10^{-8} m^3/sec or 3.2-320 L/m^2 per year at a Darcian velocity of 3.2-320×10^{-3} m/yr or at a linear average velocity of about 1.1-110 cm/year assuming a porosity of 0.3. Since clay pore water contains approximately 20 mg/L of DOC, the average flux of DOC would bring approximately 0.063-6.3 g/m^2 of carbon per year, which is equivalent to 5.3-530×10^{-3} moles of C/m^2/year. Therefore, assuming this range of flux magnitudes over the past 20 years to reflect the trend in deep pumping (the cone of depression in the area started developing at least 20 years ago and the vertical hydraulic head difference has been 1 m or greater since at least 2011, Supplementary Fig. 5), a total of 0.1-10 moles of clay-derived carbon per square meter would have been advected into the upper pre-Holocene aquifer and would have traveled 0.2-2 m into the aquifer (disregarding retardation of DOC advection).

To evaluate the impact of diffusive DOC flux in the geologic past, a negligible lateral as well as vertical head gradient is assumed under the pre-pumping conditions that allowed for a stagnant aquifer characterized by sluggish groundwater flow system. It is also assumed that the thick clay aquitard overlying the pre-Holocene aquifer contained elevated levels of DOC in the past, consistent with today's concentration gradient across the aquifer-aquitard interface. These simplified assumptions allow us to disregard the advective groundwater flow component and rely solely on the diffusive flux of DOC from the clay aquitard to the pre-Holocene aquifer based on Fick's first law: $J = -D \frac{\delta C}{\delta x} n$; where, D is the diffusivity of DOC; $\frac{\delta C}{\delta x}$ is the DOC concentration gradient across the aquitard-aquifer interface, and n is the effective porosity of clay. Using a diffusivity value for acetate of 0.009 m^2/year [14] and a DOC gradient $(\frac{\delta C}{\delta x})$ of 0.29 mM/m, calculated at the aquifer-aquitard interface at location M-Middle for a difference in DOC concentration of 17 mg C/L spanning a half of the thickness of the 10 m clay layer (Figure 3g), the DOC flux from the aquitard to the underlying aquifer, J, can be estimated for a range of clay porosity values. Assuming a range of clay porosity from 0.1 to 0.5, the diffusive flux is $0.2\text{-}1\times10^{-3}$ mol C/m^2 per year. The thick grey aquitard in the study area was dated on average 7,800±1,200 years based on 8 radiocarbon measurements at 4 locations (Supplementary Table 1). If reducing conditions were initiated after the deposition of shallow Holocene deposits ~6,000 years ago and today's DOC concentration gradient across the aquitard-aquifer interface existed 5,000 years ago, the total flux of DOC (after 5,000 years) into the upper pre-Holocene aquifer based on Fickian diffusion would be 1-5 mol C/m^2. This amount falls within the wider range of estimates for the advective flux of DOC into the upper portion of the pre-Holocene aquifer driven by pumping over the past 50 years.

How much Fe oxide can be reduced by the mid-range DOC input estimate of 1 mol C/m^2, irrespective of the transport process and duration? For comparison, we estimate the amount of Fe oxide reduction needed to turn oxidized orange sediment grey as an upper limit to the amount of DOC input required to release As in the pre-Holocene aquifer. Assuming an aquifer particle density of 2650 kg/m^3 and a porosity of 0.3, the sediment mass in a cubic meter of aquifer is 1855 kg. The average HCl-leachable Fe concentration for both orange and grey sands in the pre-Holocene aquifer is 5.6 g/kg (sample n = 28), which is roughly a half of the total Fe concentration of approximately 10 g/kg measured in these sands by X-ray fluorescence (Supplementary Fig. 2). Acid leachable Fe(II)/Fe measured in these sands ranges between 0.2 and 0.9, with the average Fe(II)/Fe in the grey sand of 0.5 (n = 17) and in the orange sand of 0.3 (n = 11). To turn 1 m^3 of the orange sand grey, therefore, considering a reduction of 20% solid phase Fe from Fe^{3+} to Fe^{2+} (i.e., 1.1 g/kg Fe), a total of 2.1 kg or 37 moles of Fe^{3+} must be reduced. The DOC input of 1 mol C/m^2 is enough to reduce 4 moles of solid phase Fe^{3+} assuming that 4 moles of Fe^{2+} is produced by the reduction of 4 moles of Fe^{3+} in the expense of 1 mole of DOC [53]. Therefore, the estimated amount of reactive DOC input would be able to change the color of a ~10 cm uniform layer of aquifer sand.

Even with an order of magnitude higher diffusive or advective flux of reactive carbon into the upper pre-Holocene aquifer, the layer of sand changing color by these processes alone would have been well below the 10-15 m thickness of grey sand underlying the clay in the

study area (Fig. 2c and Supplementary Fig.1). However, the area of contact between the clay and the upper pre-Holocene aquifer is large and spans multiple depths, as the elevation-corrected depth of the interface between the two layers varies by as much as 5-10 m among individual locations over short horizontal distances of 20-100 m (Supplementary Fig. 1). Such a large area of contact could greatly aid the processes of advection and diffusion of clay pore water into the aquifer. The subsequent dispersion of clay pore water and DOC by horizontal groundwater flow within the aquifer cannot be ignored, especially under the current lateral hydraulic head gradient in the pre-Holocene aquifer of approximately 0.002 (a lateral hydraulic head change of approximately 0.2 m over a distance of 100 m, Figure 3a). Combined with lateral flow and exchange of groundwater, either form of DOC input process (advection or diffusion) along the variable-depth interface could therefore have converted a much thicker portion of the pre-Holocene aquifer sand.

The amount of Fe reduction estimated above (4 moles in 1 m^3 of aquifer sand) is sufficient to raise Fe and As concentration to the levels observed in the upper pre-Holocene aquifer today (Fig. 3e,f). Assuming an aquifer porosity of 0.3 and that 1-5% of reduced Fe is released to the dissolved phase [7,20] over a uniform, 10 m thick square meter column of the aquifer, the reduction of 4 moles of Fe^{3+} to 4 moles of Fe^{2+} would result in dissolved Fe concentration of 0.7-3.7 mg/L in groundwater. Additionally, assuming that the solid-phase Fe contains 5.5% As by molar ratio [21] which is released to groundwater concurrently with the reduced Fe, the resulting As concentration in groundwater would be 55-275 μg/L. These calculations assume the in-situ release of Fe and As by reduction within the upper pre-Holocene aquifer and ignore the possible direct Fe and As contributions from the clay pore water transport into the aquifer (albeit subject to sorptive retardation), as well as the effects of Fe or As incorporation into rearranged Fe mineral phases such as magnetite [7,20].

Supplementary Note 6

Non-linear relationship between Fe oxide reduction and groundwater As: There is general agreement that the microbial reduction of Fe oxides within aquifer sediments is a key factor regulating the release of geogenic As to groundwater in anaerobic aquifers [17,22]. The available data indicate, however, that the relationship between dissolved As and the leachable Fe(II)/Fe ratio in aquifer sands (or an element of diffuse spectral reflectance of aquifer sands as a proxy thereof) is far from linear [7,23]. For Fe(II)/Fe ratios ranging from 0 to 0.5, which correspond to sediment color ranging from orange to grey-brown, groundwater As concentrations in Bangladesh generally remain <10 μg/L. When Fe(II)/Fe ratios exceed 0.5, groundwater As concentrations can range anywhere between 50 and 500 μg/L. This needs to be taken into account when interpreting the few detailed time series of groundwater As that are available, including those from the present study site. The implication is that a steady flux of reactive carbon reaching an aquifer may not have a detectable impact on groundwater As for an extended period until the Fe(II)/Fe threshold is reached. When a steady reactive carbon influx triggered or enhanced by Dhaka pumping causes detectable release of As to groundwater may depend on highly localized conditions, including the degree of reduction of Fe oxides that prevailed before the onset of perturbation, and therefore may not be synchronous at different locations.

Supplementary Note 7

Other Modes of Delivery of Clay Pore Water into Aquifer: In addition to the Darcian flow through the clay layer, diffusion along the contact with aquifer sediments, and dispersion by groundwater flow within the aquifer, a large volume of clay pore water carrying DOC and As can be delivered by squeezing of clay during subsidence processes. Recent reports from the Mekong delta in Vietnam [24] and from the Central Valley of California [16] linked the widespread As contamination of massively pumped confined aquifers to the land subsidence. Massive groundwater pumping lowers hydraulic head (i.e. pore pressure) in aquifers, which increases the effective stress on aquifer materials, causing the aquifer and aquitard (clay layers) to compact. A major portion of land subsidence comes from the compaction of clays due to their weaker geomechanical properties and significant porosity, which in the process drains stored water in the aquitard and delivers clay pore water with As and organic matter into the aquifer [16,24]. Near Dhaka, land subsidence can reach up to 1.2 cm/yr [25], thus it could have an impact on leaching of the labile organics stored in clay layers into the aquifers. Besides the clay squeezing due to large-scale pumping depressurization in the recent years, clay pore water can also ooze into the aquifer due to seasonal loading and unloading (i.e. relaxing and contraction) of the clay layers caused by monsoonal cycles [15]; this process would have operated for millennia and the recent subsidence due to large-scale groundwater extraction would only exacerbate an existing problem when aquifer is in proximity of a clay layer loaded with reactive organic matter and arsenic.

Supplementary references

1 Aeschbach-Hertig, W., Peeters, F., Beyerle, U. & Kipfer, R. Interpretation of dissolved atmospheric noble gases in natural waters. Water Resources Research 35, 2779-2792, doi:10.1029/1999wr900130 (1999).

2 Stute, M. & Schlosser, P. in Environmental tracers in subsurface hydrology (eds P. G. Cook & A. L. Herczeg) 349-377 (Kluwer, 1999).

3 Stute, M. et al. Hydrological control of As concentrations in Bangladesh groundwater. Water Resources Research 43, doi:W09417 Artn w09417 (2007).

4 Ekwurzel, B. et al. Dating of shallow groundwater - comparison of the transient tracers H-3/He-3, chlorofluorocarbons, and Kr-85. Water Resources Research 30, 1693-1708, doi:10.1029/94wr00156 (1994).

5 Stute, M. et al. Tritium/He-3 dating of river infiltration: An example from the Danube in the Szigetkoz area, Hungary. Ground Water 35, 905-911, doi:10.1111/j.1745-6584.1997.tb00160.x (1997).

6 Stute, M. in Groundwater Arsenic Contamination in the Bengal Delta Plain of Bangladesh, Proceedings of the KTH-Dhaka University Seminar. (ed G. et al. Jacks) 109-117 (KTH Spec. Publ. TRITA-AMI Rep. 3084).

7 Horneman, A. et al. Decoupling of As and Fe release to Bangladesh groundwater under reducing conditions. Part 1: Evidence from sediment profiles. Geochimica Et Cosmochimica Acta 68, 3459-3473, doi:10.1016/j.gca.2004.01.026 (2004).

8 Nickson, R. T., McArthur, J. M., Ravenscroft, P., Burgess, W. G. & Ahmed, K. M. Mechanism of arsenic release to groundwater, Bangladesh and West Bengal. Applied Geochemistry 15, 403-413, doi:10.1016/s0883-2927(99)00086-4 (2000).

9 Postma, D. et al. Arsenic in groundwater of the Red River floodplain, Vietnam: Controlling geochemical processes and reactive transport modeling. Geochimica Et Cosmochimica Acta 71, 5054-5071, doi:10.1016/j.gca.2007.08.020 (2007).

10 Berg, M. et al. Hydrological and sedimentary controls leading to arsenic contamination of groundwater in the Hanoi area, Vietnam: The impact of iron-arsenic ratios, peat, river bank deposits, and excessive groundwater abstraction. Chemical Geology 249, 91-112, doi:10.1016/j.chemgeo.2007.12.007 (2008).

11 McArthur, J. M., Ravenscroft, P., Safiulla, S. & Thirlwall, M. F. Arsenic in groundwater: Testing pollution mechanisms for sedimentary aquifers in Bangladesh. Water Resources Research 37, 109-117 (2001).

12 Buschmann, J. & Berg, M. Impact of sulfate reduction on the scale of arsenic contamination in groundwater of the Mekong, Bengal and Red River deltas. Applied Geochemistry 24, 1278-1286, doi:10.1016/j.apgeochem.2009.04.002 (2009).

13 Neumann, R. B. et al. Anthropogenic influences on groundwater arsenic concentrations in Bangladesh. Nature Geoscience 3, 46-52, doi:10.1038/ngeo685 (2010).

14 McMahon, P. B. & Chapelle, F. H. Microbial production of organic acids in aquitard sediments and its role in aquifer geochemistry. Nature 349, 233-235, doi:10.1038/349233a0 (1991).

15 Planer-Friedrich, B. et al. Organic carbon mobilization in a Bangladesh aquifer explained by seasonal monsoon-driven storativity changes. Applied Geochemistry 27, 2324-2334, doi:https://doi.org/10.1016/j.apgeochem.2012.08.005 (2012).

16 Smith, R., Knight, R. & Fendorf, S. Overpumping leads to California groundwater arsenic threat. Nature Communications 9, doi:http://doi.org/10.1038/s41467-018-04475-3 (2018).

17 Fendorf, S., Michael, H. A. & van Geen, A. Spatial and Temporal Variations of Groundwater Arsenic in South and Southeast Asia. Science 328, 1123-1127, doi:10.1126/science.1172974 (2010).

18 Harvey, C. F. et al. Arsenic mobility and groundwater extraction in Bangladesh. Science 298, 1602-1606 (2002).

19 Zheng, Y. et al. Geochemical and hydrogeological contrasts between shallow and deeper aquifers in two villages of Araihazar, Bangladesh: Implications for deeper aquifers as drinking water sources. Geochimica Et Cosmochimica Acta 69, 5203-5218 (2005).

20 van Geen, A. et al. Decoupling of As and Fe release to Bangladesh groundwater under reducing conditions. Part II: Evidence from sediment incubations. Geochimica Et Cosmochimica Acta 68, 3475-3486, doi:10.1016/j.gca.2004.02.014 (2004).

21 Burnol, A. et al. Decoupling of arsenic and iron release from ferrihydrite suspension under reducing conditions: a biogeochemical model. Geochemical transactions 8, 12, doi:10.1186/1467-4866-8-12 (2007).

22 BGS/DPHE. Arsenic contamination of groundwater in Bangladesh, BGS echnical Report WC/00/19 (Catalogue No. BGS Technical Report WC/00/19, British

Geological Survey and Department of Public Health Engineering, Keyworth, UK, 2001).
23 van Geen, A. et al. Flushing history as a hydrogeological control on the regional distribution of arsenic in shallow groundwater of the Bengal Basin. Environmental Science & Technology 42, 2283-2288, doi:10.1021/es702316k (2008).
24 Erban, L. E., Gorelick, S. M., Zebker, H. A. & Fendorf, S. Release of arsenic to deep groundwater in the Mekong Delta, Vietnam, linked to pumping-induced land subsidence. Proceedings of the National Academy of Sciences of the United States of America 110, 13751-13756, doi:10.1073/pnas.1300503110 (2013).
25 Steckler, M. S. et al. Modeling Earth deformation from monsoonal flooding in Bangladesh using hydrographic, GPS, and Gravity Recovery and Climate Experiment (GRACE) data. Journal of Geophysical Research-Solid Earth 115, doi:10.1029/2009jb007018 (2010).
26 Khan, M. R. et al. Megacity pumping and preferential flow threaten groundwater quality. Nature Communications 7, 12833, doi:10.1038/ncomms12833 https://www.nature.com/articles/ncomms12833 - supplementary-information (2016).
27 Knappett, P. S. K. et al. Vulnerability of low-arsenic aquifers to municipal pumping in Bangladesh. Journal of Hydrology 539, 674-686, doi:10.1016/j.jhydrol.2016.05.035 (2016).
28 Bethke, C. M. & Johnson, T. M. Groundwater age and groundwater age dating. Annual Review of Earth and Planetary Sciences 36, 121-152 (2008).
